# Functional Analysis of Novel *alkB* Genes Encoding Long-Chain *n*-Alkane Hydroxylases in *Rhodococcus* sp. Strain CH91

**DOI:** 10.3390/microorganisms11061537

**Published:** 2023-06-09

**Authors:** Wei Xiang, Shan Hong, Yanfen Xue, Yanhe Ma

**Affiliations:** State Key Laboratory of Microbial Resources, Institute of Microbiology, Chinese Academy of Sciences, Beijing 100101, China

**Keywords:** alkane degradation, bioremediation, hypothetical *alkB* gene, alkane 1-monooxygenase

## Abstract

*Rhodococcus* sp. strain CH91 is capable of utilizing long-chain *n*-alkanes as the sole carbon source. Two new genes (*alkB1* and *alkB2*) encoding AlkB-type alkane hydroxylase were predicted by its whole-genome sequence analysis. The purpose of this study was to elucidate the functional role of *alkB1* and *alkB2* genes in the *n*-alkane degradation of strain CH91. RT-qPCR analyses revealed that the two genes were induced by *n*-alkanes ranging from C16 to C36 and the expression of the *alkB2* gene was up-regulated much higher than that of *alkB1*. The knockout of the *alkB1* or *alkB2* gene in strain CH91 resulted in the obvious reduction of growth and degradation rates on C16-C36 *n*-alkanes and the *alkB2* knockout mutant exhibited lower growth and degradation rate than the *alkB1* knockout mutant. When gene *alkB1* or *alkB2* was heterologously expressed in *Pseudomonas fluorescens* KOB2Δ1, the two genes could restore its alkane degradation activity. These results demonstrated that both *alkB1* and *alkB2* genes were responsible for C16-C36 *n*-alkanes’ degradation of strain CH91, and *alkB2* plays a more important role than *alkB1*. The functional characteristics of the two *alkB* genes in the degradation of a broad range of *n*-alkanes make them potential gene candidates for engineering the bacteria used for bioremediation of petroleum hydrocarbon contaminations.

## 1. Introduction

Long-chain *n*-alkanes are part of the main components of petroleum. The biodegradation of long-chain *n*-alkanes by bacteria has attracted considerable attention due to its potential for bioremediation of petroleum contamination and microbial-enhanced oil recovery [1,2,3,4]. Several bacteria including *Acinetobacter*, *Pseudomonas*, *Dietzia*, *Rhodococcus*, *Geobacillus*, etc. have been reported to be able to degrade long-chain *n*-alkanes [5,6]. The biodegradation of long-chain *n*-alkanes in these bacteria is usually initiated by the terminal oxidation of *n*-alkane to the corresponding alkanol, which is the key step in the *n*-alkane degradation pathway and catalyzed by an alkane hydroxylase. Several alkane hydroxylase systems involved in long-chain *n*-alkane degradation have been identified, including integral-membrane non-heme diiron monooxygenase (AlkB), cytochrome P450 monooxygenase, flavin-binding alkane hydroxylase (AlmA), and flavin-dependent alkane monooxygenase (LadA) [5,7].

The alkB-type alkane hydroxylase system of *Pseudomonas putida* GPo1 is the best-characterized system for alkane degradation [8,9]. It catalyzes the initial terminal oxidation of *n*-alkane in the form of a three-component complex, which consists of a non-heme integral-membrane alkane hydroxylase (AlkB), two rubredoxins (AlkF and AlkG), and a rubredoxin reductase (AlkT). Among the three-component complexes, rubredoxin and rubredoxin reductase are essential electron transfer components required for alkane hydroxylation by AlkB [8,10]. Similar to *P. putida* GPo1, *Acinetobacter* sp. ADP1 and 6A2 also catalyze the initial oxidation of C12–C18 *n*-alkanes via a three-component alkane hydroxylase system, which comprises AlkM (alkane monooxygenase), RubA (rubredoxin), and RubB (rubredoxin reductase) [11,12,13]. Whyte et al. identified two three-component alkane hydroxylase systems (AlkB1 and AlkB2) in *Rhodococcus erythropolis*, which are partially responsible for the initial oxidation of C12–C16 *n*-alkanes [14]. The *alkB* homologs of *M. tuberculosis* and *P. aeruginosa* were also shown to oxidize alkanes ranging from C10 to C16 [15]. The three-component alkane hydroxylase system containing alkane 1-monooxygenase (AlkB), rubredoxin (RubA), and rubredoxin reductase (RubB) is often involved in alkane hydroxylation [5,7]. Other types of alkane hydroxylation mechanisms have also been reported. Cytochrome P450 hydroxylase usually mediates the oxidation of short- and medium-chain alkanes (C5–C16). It has been reported to catalyze the terminal oxidation of *n*-alkane in *Acinetobacter* sp. EB104, *Mycobacterium* sp. HXN-1500, and *Alcanivorax dieselolei* B-5 [16]. AlmA-type alkane hydroxylase was first identified in *Acinetobacter* sp. DSM 17874 and oxidize C20 to >C32 *n*-alkanes [17]. Its homologs also have been identified in several other long-chain *n*-alkane-degrading strains, including *Acinetobacter pittii* SW-1, *Acinetobacter* sp. M1, *Alcanivorax borkumensis* SK2, and *Pseudomonas aeruginosa* DN1 [3,18]. LadA-type alkane monooxygenase was first reported in *Geobacillus thermodenitrificans* NG80-2 and could oxidize C15–C36 *n*-alkanes into primary alcohols. The enzyme is a two-component flavin-dependent oxygenase belonging to the bacterial luciferase family of proteins [19].

The AlkB-type alkane hydroxylases have been widely found in alkane-degrading bacteria by gene cloning, PCR detection, and genome sequencing. They are quite divergent and have different substrate ranges. The well-characterized AlkB-type alkane hydroxylases are usually reported to be involved in the metabolism of *n*-alkanes up to C16 [5,7]. Only a few AlkB-type alkane hydroxylases were experimentally confirmed in the degradation of *n*-alkanes longer than C18 [20,21]. The contributions of AlkB-type alkane hydroxylases to the long-chain alkane degradation are still far from clear. In a previous study, we isolated a novel *Rhodococcus* strain, CH91, with the ability to degrade long-chain *n*-alkanes, and two new *alkB* genes encoding alkane hydroxylase were annotated in the genome of this strain [22]. The enzymes encoded by the two *alkB* genes of strain CH91 had a lower identity with the well-characterized AlkB type alkane hydroxylases but showed significant similarity with the homologs from *R. rhodochrous* NCTC 10210, *R. pyridinivorans* DSM 44555, *R. biphenylivorans* TG9, and *R. gordoniae* NCTC13296, which belong to the *R. rhodochrous* subclade [22]. All of these homologs from *R. rhodochrous* subclade strains were annotated solely according to sequence homology, but their functions have not yet been experimentally characterized. As every AlkB enzyme is unique and showed different substrate ranges, elucidating its biochemical characterization is important for understanding the metabolic pathway of alkane degradation and realizing its biotechnological potential. In this study, we clarified the contribution of the two *alkB* genes to the long-chain alkane degradation of *Rhodococcus* sp. strain CH91 by constructing *alkB* gene knockout mutants and heterologously expressing the two *alkB* genes, together with analyzing the genetic characteristics and transcriptional expression profile of *alkB* genes.

## 2. Materials and Methods

### 2.1. Bacterial Strains and Cultivation

The bacterial strains and plasmids used in this study are listed in Supplementary Table S1. *Rhodococcus* sp. strain CH91 and its mutant derivatives were grown at 37 °C in Luria-Bertani (LB) medium or MD878 basal medium supplemented with *n*-alkanes or succinate as a sole carbon source [22]. *P. fluorescens* KOB2∆1 and its recombinants were grown at 30 °C in LB medium or E2 medium [23] supplemented with *n*-alkanes as sole carbon sources. *E. coli* DH5α was used for plasmid construction and grown at 37 °C in an LB medium. *E. coli* strains were transformed with plasmid DNA via calcium-dependent transformation [24]. When necessary, appropriate antibiotics were used in the medium: ampicillin (at the final concentration of 100 µg mL^−1^), kanamycin (50 µg mL^−1^), chloramphenicol (30 µg mL^−1^), and gentamicin (100 µg mL^−1^ for *P. fluorescens* and strain CH91, 10 µg mL^−1^ for *E. coli*).

### 2.2. Growth and Alkane Degradation Assays

To determine the utilization of various *n*-alkanes, strain CH91 and its mutant derivatives were grown in an MD878 medium supplemented with various *n*-alkanes as the sole carbon source. Different alkanes were first dissolved in octane and then individually added to the medium at a final concentration of 0.4% (*v*/*v*) for the liquid alkane (C16–C20) at 37 °C or 0.1% (*w*/*v*) for the solid alkane (C24–C36). The utilization of mixed *n*-alkanes was also tested with a final concentration of 1% (*v*/*v*) *n*-alkane mixture as the sole carbon source. The un-inoculated cultures were used as controls. The influence of the solvent octane on the growth of the cells was measured by using 1% (*v*/*v*) octane as the sole carbon source. All cultures were incubated on a shaker at 150 rpm at 37 °C for 10 days for C16–C20 carbon sources or 21 days for C24–C36 carbon sources or 14 days and 24 days for the *n*-alkane mixture. All the experiments were repeated in triplicate. The growth was examined by measuring the optical density at 600 nm and the alkane degradation was determined by analysis of the residual *n*-alkane by GC-MS chromatography. 

The *n*-alkane mixture used in this study contained 5% (*v*/*v*) C16 and 1% (*w*/*v*) each of C18, C20, C24, C26, C28, C30, C32, and C36 *n*-alkanes dissolved in octane. Hydrocarbons such as hexane (C6), octane (C8), hexadecane (C16), octadecane (C18), eicosane (C20), tetracosane (C24), hexacosane (C26), octacosane (C28), triacontane (C30), dotriacontane (C32) and hexatriacontane (C36) were purchased from Macklin (Ghaziabad, India).

### 2.3. Analytical Methods

As the strain formed clumps with alkane when growing on alkane and the cells were difficultly spun down, the cultures grown on *n*-alkanes were firstly treated by adding *n*-hexane to dissociate the cells from a long-chain alkane. The entire culture was extracted twice with 1/5 volume of *n*-hexane following the addition of dioctyl phthalate as an internal standard. The mixtures were centrifuged at 10,000× *g* for 20 min at room temperature. The cell pellet was re-suspended with 1/2 volume of buffer solution and its optical density was measured at 600 nm as the growth of strain. The hexane layer was harvested and used to analyze the amount of residual alkanes using GC-MS (Agilent (Santa Clara, CA, USA) 5977A MS and 7890B GC equipped with FID detector) attached to an Agilent HP-5MS capillary column [22]. The FID detector was used for quantitative analysis. The degradation ratio of *n*-alkanes was calculated with the equation R (%) = (Co − Cx)/Co × 100, where R, Co, and Cx represent the *n*-alkane degradation ratio, the residual *n*-alkane concentration in the un-inoculated culture, and the concentration in the inoculated culture, respectively. All analyses were carried out with three replicates, and the values are presented as mean values ± standard deviations (SD). Data were analyzed statistically using a two-tailed T-TEST, and *p*-values of 0.05 or less were considered statistically significant. Calculations and graphics were performed using GraphPad Prism 8. 

### 2.4. Reverse Transcription and Real-Time Quantitative PCR (RT-qPCR)

To investigate the induction of *alkB1* and *alkB2* by different *n*-alkanes, strain CH91 was cultured to the mid-exponential phase of growth in MD878 medium supplemented with 0.5% succinate or 0.1% (*w*/*v*) different *n*-alkanes as sole carbon sources. Cells were collected by centrifugation at 10,000× *g* for 10 min at 4 °C. Total RNA was extracted using Bacterial RNAprep Pure Kit (Tiangen, Beijing, China). Reverse transcription was performed using StarScript III All-in-one RT-PCR Kit (GenStar, Beijing, China). RT-qPCR analysis was conducted using a 2xRealStar Fast SYBR qPCR mix (GenStar, Beijing, China) on a CFX96 Real-Time System (BIO-RAD, Hercules, CA, USA). 16S rRNA gene was used as an internal control for normalization. A control reaction without reverse transcriptase was conducted to verify the absence of genomic DNA. All of the experiments were performed according to the instructions from the manufacturers of the reagents or instruments. Relative quantities were calculated using the 2^−ΔΔCt^ method [25] with the succinate carbon source as the control. All experiments were performed in triplicate and the means and standard deviations were calculated. The primers used for RT-qPCR were designed with the Primer Premier 5.0 software package (Premier Biosoft Intl., Palo Alto, CA, USA) and are listed in Supplementary Table S2. 

### 2.5. Construction of alkB Gene Knockout Mutants

To construct *alkB* gene knockout mutants, a CRISPR/Cas9-mediated triple-plasmid genome editing system was employed as previously described [26]. Briefly, wild-type strain CH91 was transformed with the plasmid pNV-Pa2-Cas9 and then made competent for the introduction of pRCTc-Pa2-Che9c60&61. The subsequent CH91 (Cas9+Che9c60&61) was transformed with 1 μg of the pBNVCm-sgRNA series and 1 μg of linear donor dsDNA. Cells were spread on LB+ 1% pyruvate plates containing 50 μg  mL^−1^ kanamycin, 10 μg  mL^−1^ tetracycline, and 25 μg  mL^−1^ chloramphenicol and then incubated at 28 °C for 3–5 days to obtain target strains. Knockout of the genes was confirmed by PCR amplification and sequencing of the amplified regions flanking the deletions. The genes *alkB1* and *alkB2* were individually knocked out by means of the procedure outlined above. The *alkB*-sgRNA was designed on the websites http://grna.ctegd.uga.edu/ and http://www.oligoevaluator.com/LoginServlet accessed on 15 September 2021. The primers used for gene knockout in strain CH91 are listed in Supplementary Table S2.

### 2.6. Heterologous Expression of alkB Genes

*P. fluorescens* KOB2Δ1 is an *alkB1* deletion derivative of *P. fluorescens* CHA0 and is usually used to assess the activities of alkane hydroxylases via growth complementation by restoring alkane degradation through heterologous expression of *alkB* ortholog genes [27,28]. To confirm the alkane hydroxylase activity of *alkB* genes from *Rhodococcus* sp. strain CH91, both *alkB* genes were also heterologously expressed and evaluated in *P. fluorescens* KOB2Δ1. The genes *alkB1* and *alkB2* were individually amplified by PCR using *Rhodococcus* sp. CH91 chromosomal DNA as a template and cloned into pCom8 plasmid at *Nde*I and *Hind*III sites using ClonExpress^®^ Ultra One Step Cloning Kit (Vazyme, Nanjing, China) by Gibson assembly method [29]. The primers used for PCR amplification of *alkB* genes were designed based on the genome sequence of strain CH91 and listed in Supplementary Table S2. The nucleotide sequences of the pCom8-*alkB* plasmids were confirmed to be correct by sequencing using an ABI 3730 automated DNA sequencer (Applied Biosystems, Foster City, CA, USA). Successful plasmids were transformed into *P. fluorescens* KOB2∆1 via electroporation as described before [30]. *P. fluorescens* KOB2Δ1 recombinants containing pCom8 (as negative control) and pCom8-*alkB* plasmids were grown in an E2 medium [23] supplemented with 1% *n*-alkane mixture as sole carbon sources. The cultures were incubated under shaking conditions (150 rpm) at 30 °C for 21 days. The growth was examined by measuring the optical density at 600 nm and the alkane degradation was determined by analysis of the residual *n*-alkane by GC-MS chromatography. The un-inoculated culture was used as a control. All the experiments were repeated in triplicate. 

## 3. Results and Discussion

### 3.1. Genetic Characteristics of alkB Genes in Rhodococcus *sp.* CH91

**Figure 1:**
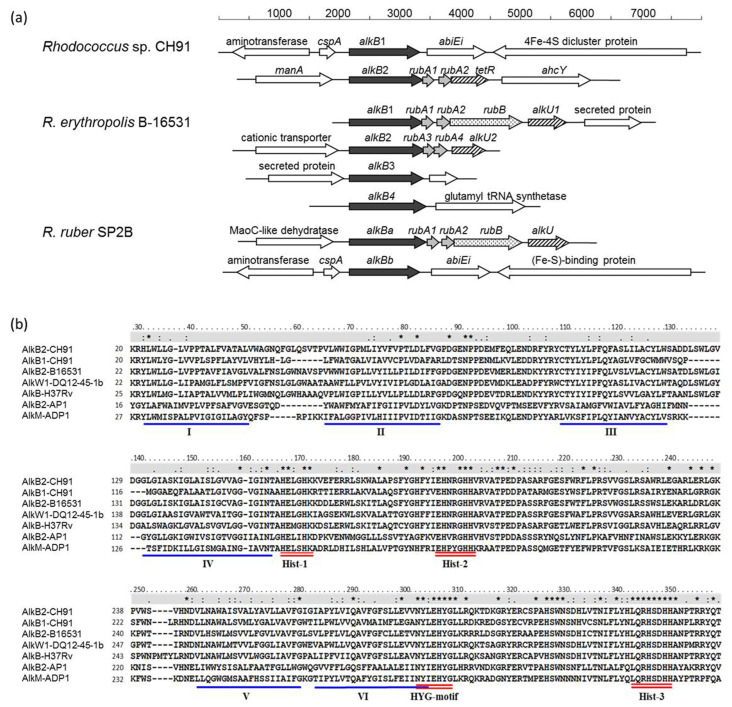
(**a**) Representative gene organizations of *alkB* gene regions from *Rhodococcus* strains. Similar shading patterns of the arrows represent similar functions: black arrow, *alkB* gene-encoding alkane hydroxylase; grey arrow, *rubA*-encoding rubredoxin; vertically striped arrow, *rub*-encoding rubredoxin reductase; diagonally striped arrow, *tetR*/*alkU* transcriptional regulator protein; open arrow, other genes; (**b**) Part of the multiple sequence alignments of *Rhodococcus* sp. CH91 AlkBs with some other known alkane hydroxylases. Conserved histidine boxes (Hist-1, Hist-2, and Hist-3) and the HYG motif are underlined by a red double line. The six putative transmembrane helices are underlined by a blue single line and marked by Roman numerals. The line above the alignment indicates the conserved positions: “*”, a fully conserved amino acid residue in all aligned alkane hydroxylases; “:”, a conserved strong group positions; “.”, a conserved weaker group position. AlkB2-B16531, alkane hydroxylase from *R. erythropolis* B-16531 (accession number CAC37038); AlkW1-DQ12-45-1b, *Dietzia* sp. DQ12-45-1b (AEM66514); AlkB-H37Rv, *Mycobacterium tuberculosis* H37Rv (NP_217769); AlkB2-AP1, *Alcanivorax borkumensis* AP1 (AJ577851); AlkM-ADP1, *Acinetobacter* sp. ADP1 (AJ002316).

*Rhodococcus* sp. strain CH91 is capable of utilizing *n*-alkanes with carbon chain lengths ranging from C16 to C36 as a sole carbon source, and no growth on C8, C12, and C40 was observed. Its complete genome was previously sequenced in our lab and two *alkB*-type alkane hydroxylase genes (*alkB1* and *alkB2*) were predicted to be responsible for the first step of the *n*-alkane degradation pathway [22]. The open reading frame (ORF) analysis of the *alkB* gene loci revealed that the genetic arrangement of the two *alkB* gene regions had different organizations. The *alkB2* gene region contained an operon-like structure that had four genes encoding an alkane hydroxylase (AlkB2), a couple of rubredoxins (RubA1 and RubA2), and a TetR transcriptional regulator (Figure 1a). Moreover, the *alkB2* and *rubA1* genes had 3′-end–5′-end GTGA sequence overlaps, and the *rubA1* and *rubA2* genes had 3′-end–5′-end ATGA sequence overlaps. The gene organization is quite similar to the *alkB2* gene cluster previously demonstrated in other *Rhodococcus* strain genomes [14]. The *alkB1* gene region contained a separate alkane hydroxylase homolog and was not flanked by rubredoxin or rubredoxin reductase genes (Figure 1a). The genes upstream *alkB1* encoded a cold-shock protein and an aminotransferase. The genes downstream *alkB1* encoded AbiEi family antitoxin domain-containing protein and 4Fe-4S dicluster domain-containing protein. The ORF organization of the *alkB1* gene and the surrounding region was not like that of the *alkB3* and *alkB4* gene regions of *R. erythropolis* B-16531 and Q15 [14], but quite similar to that of the *alkBb* gene regions of *R. ruber* NBRC 15591 and SP2B as well as that of *R. rhodochrous* NCTC 10210 and NBRC 16069, *R. pyridinivorans* TG9 and DSM 44555, *R. gordoniae* NCTC13296 and *R. coprophilus* NCTC10994, which belong to the *R. rhodochrous* subclade.

Multiple sequence alignments of the AlkB1 and AlkB2 amino acid sequences were performed with other published AlkB sequences using the ClustalW algorithm in MEGA7 software (version 7.0) [31]. As shown in Figure 1b, both AlkB1 and AlkB2 proteins possessed eight histidine residues within three His boxes (Hist1, HELGHK; Hist2, EHNRGHH; and Hist3, LQRHSDHHA) and an HYG motif (NYLEHYGL), which are highly conserved in non-heme iron integral membrane alkane hydroxylases and required for catalytic activity [14,32]. In addition, both AlkB1 and AlkB2 enzymes possessed the six transmembrane helices, which are also conserved in all integral membrane alkane hydroxylase [14,33]. These structural characteristics suggest that AlkB1 and AlkB2 are membrane-bound alkane hydroxylases and might be responsible for long-chain *n*-alkane degradation in strain CH91.

### 3.2. Transcriptional Expression of n-Alkane Hydroxylase Genes in Rhodococcus *sp.* Strain CH91

**Figure 2:**
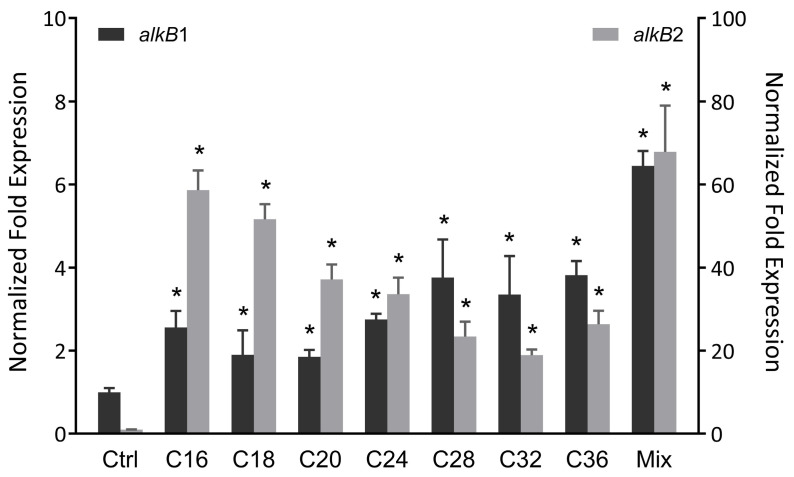
Quantitative RT-PCR analysis of the transcription levels of *alkB1* and *alkB2* in strain CH91 grown on *n*-alkanes C16 to C36 in length. 16S rRNA as an internal control for normalization. The Left Y axis represents the expression level of *alkB1*, and the right Y axis represents the expression level of *alkB2*. Mix means *n*-alkane mixture containing C16, C18, C20, C24, C26, C28, C30, C32 and C36. The data represent mean  ±  SD. *, *p*  <  0.05 compared to succinate control (*n*  =  3/group).

To investigate the *alkB* gene expression profile in strain CH91, RT-qPCR was performed to analyze the expression level of *alkB1* and *alkB2* genes in the presence of different *n*-alkanes ranging from C16 to C36. As shown in Figure 2, both the *alkB* genes were induced by the tested long-chain *n*-alkanes and showed obviously higher expression in the presence of the *n*-alkane mixture than in the individual *n*-alkane. The expression of *alkB2* was remarkably induced by all of the *n*-alkanes tested, the largest increase (58-fold) was observed with C16, and the lower expression level was induced normally with the longer chain length of *n*-alkane. The expression of *alkB1* was induced about 2-fold in the presence of C16–C24 and up-regulated to about 3.5-fold in the presence of C28–C36, indicating the presence of the different regulatory systems for *alkB1* response to different *n*-alkane substrate range. Moreover, the different patterns of the transcriptional expression induced by *n*-alkanes between *alkB1* and *alkB2* suggest a different regulatory mechanism for *alkB1* and *alkB2*. The above data supported that *alkB1* and *alkB2* are involved in C16–C36 *n*-alkane degradation of strain CH91, and *alkB2* plays the predominant role in *n*-alkane degradation for strain CH91. The different induction manners in response to the chain length of *n*-alkanes were also reported in other strains. For example, *Acinetobacter* sp. M-1 contains two AlkB-related alkane hydroxylases, AlkMa and AlkMb, whose expressions are controlled by different regulators. The expression of *alkMa* is induced by *n*-alkanes with chain lengths more than C22, and *alkMb* expression is preferentially induced by C16–C22 *n*-alkanes [34]. Regarding other *Rhodococcus* members, strain TMP2 has five alkane hydroxylases. The expressions of *alkB1* and *alkB2* genes are induced by C16 and pristine, whereas *alkB3*, *alkB4,* and *alkB5* genes were not affected by C16 or pristine [35]. Similarly, *R. erythropolis* PR4 possesses four alkane monooxygenases. Only *alkB1* and *alkB2* genes were highly upregulated by C16 and diesel oil. The other two *alkB* genes did not change obviously. The induction level of *alkB1* was much higher than that of *alkB2* [36]. *Pseudomonas aeruginosa* RR1, like our strain CH91, has two alkane hydroxylases. Both genes could be induced by C10–C22 *n*-alkanes. The induction level of *alkB2* is about twice as much as that of *alkB1* [37].

### 3.3. Utilization of n-Alkanes by Wild-Type Strain CH91 and Its Mutant Derivatives

**Figure 3:**
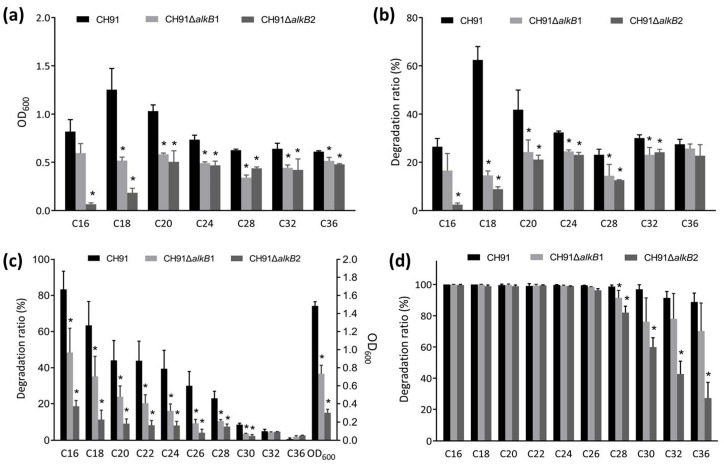
Growth and degradation of strain CH91 wild-type and mutants on different *n*-alkanes. (**a**,**b**) growth and degradation, respectively, after cultivation at 37 °C in MD878 medium supplemented with 0.4% (*v*/*v*) of C16, C18, or C20 for 10 days or 0.1% (*w*/*v*) of C24, C28, C32, or C36 as the sole carbon source for 21 days; (**c**,**d**) growth and degradation after cultivation at 37 °C for 14 days and 24 days, respectively, with the *n*-alkane mixture (contained 0.05% C16 and 0.01% each of C18, C20, C22, C24, C26, C28, C30, C32, and C36) as the sole carbon sources. Growth was indicated by the optical density at 600 nm and *n*-alkane degradation was determined by GC-MS analysis of the residual *n*-alkane. The mean values and standard deviations are shown. *, *p* < 0.05 compared with wild strain CH91 (*n* = 3/group).

To examine the possible function of the two *alkB* genes in *n*-alkane utilization, the *alkB* gene knockout mutants (CH91Δ*alkB1* and CH91Δ*alkB2*) of strain CH91 were constructed and functionally analyzed. Wild-type strain CH91 and its mutant derivatives were cultivated with different *n*-alkanes ranging in length from C16 to C36 as the sole carbon source. As shown in Figure 3a,b, wild-type strain CH91 had faster growth and higher degradation efficiency with C16, C18, or C20 *n*-alkane as the sole carbon source, while it showed some slower growth and lower degradation rate in the case of C24, C28, C32 or C36 *n*-alkane as sole carbon source. The best capability of growth and degradation for strain CH91 occurred on C18 as the sole carbon source and then on C20. When the *alkB1* or *alkB2* gene was knocked out in strain CH91, the growth and degradation efficiency were decreased to different extents in the presence of every tested *n*-alkanes, suggesting that *alkB1* and *alkB2* are responsible for C16–C36 *n*-alkane degradation in strain CH91. A significant decrease in the growth and degradation activity occurred in both the mutants in the case of C18 or C20 as the sole carbon source. When the mutants were grown in the presence of C16 as the sole carbon source, the mutant CH91Δ*alkB2* almost completely lost the growth and degradation ability, while the mutant CH91Δ*alkB1* exhibited only a slight decrease in its ability to utilize *n*-hexadecane. These results suggest that both *alkB1* and *alkB2* are essential for the utilization of C18 and C20 *n*-alkanes in strain CH91 and *alkB2* plays a key role in the *n*-hexadecane utilization of strain CH91. We tried to knock out both genes *alkB1* and *alkB2* simultaneously in strain CH91. Unfortunately, the attempt to obtain a double mutant Δ*alkB1B2* was not successful.

We also investigated the behavior of the mutants in the utilization of the long-chain *n*-alkane mixture including chain length from C16 to C36. As shown in Figure 3c, after 14 days of incubation, the growth of the mutants CH91Δ*alkB1* and CH91Δ*alkB2* decreased significantly and the mutant CH91Δ*alkB2* exhibited lower growth than that of the mutant CH91Δ*alkB1*. GC-MS analysis of residual *n*-alkanes showed that the degradation ability of the two mutants was significantly decreased for C16–C30 *n*-alkanes and the mutant CH91Δ*alkB2* had a lower degradation rate than the mutant CH91Δ*alkB1*. When the culturing time was prolonged to over 24 days (Figure 3d), the mutants CH91Δ*alkB1* and CH91Δ*alkB2* showed an obvious decrease in the degradation ability for C28–C36 *n*-alkanes, while C16–C26 were almost completely degraded by wild strain CH91 and its two mutants. These data also support the functional roles of *alkB1* and *alkB2* in the C16–C36 *n*-alkane utilization of strain CH91. Interestingly, the wild-type strain CH91 and the two mutants (CH91Δ*alkB1* and CH91Δ*alkB2*) exhibited a decrement in the degradation rate of the *n*-alkanes in sequence from C16 to C36 in the long-chain *n*-alkane mixture (Figure 3c,d). The phenomenon is different from that of the strain with individual *n*-alkane as the sole carbon source (Figure 3b). This could be explained by the supposition of the different induction mechanisms and intensity for different *n*-alkane substrates inferred from the results of RT-qPCR above.

Many strains have multiple alkane hydroxylases, each one being active on certain chain-length alkanes [3,18]. For example, *Alcanivorax borkumensis* AP1 contains two AlkB-type alkane hydroxylases, AlkB1 and AlkB2. AlkB1 oxidizes C5–C12 *n*-alkanes while AlkB2 is active on C10–C16 *n*-alkanes [38]. Park et al. identified the presence of two genes *alkB1* and *alkB2* in *Acinetobacter oleivorans* DR1. The gene *alkB1* is responsible for long-chain alkane utilization (C24–C26) and *alkB2* for medium-chain alkane (C12–C16) metabolism [21]. While our mutational analysis, consistent with our RT-qPCR data, revealed that both *alkB1* and *alkB2* genes in strain CH91 are responsible for C16–C36 *n*-alkane metabolism. A similar result was found in *Gordonia* sp. strain SoCg. The *Gordonia alkB* gene is active on a wide range of long-chain *n*-alkanes (C16–C36) [20].

### 3.4. Functional Complementation of Strain CH91 alkB Genes in P. fluorescens KOB2Δ1

**Figure 4:**
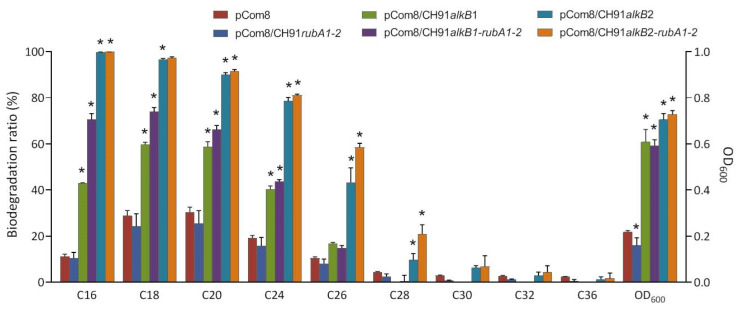
Growth and degradation of *P. fluorescens* KOB2Δ1 recombinants harboring pCom8 with CH91*alkB* genes on *n*-alkanes. Cells were grown on E2 medium supplemented with the *n*-alkane mixture (contained 0.05% C16 and 0.01% each of C18, C20, C24, C26, C28, C30, C32 and C36) as sole carbon source at 30 °C for 21 days. Growth was indicated by the optical density at 600 nm and degradation of *n*-alkane was determined by GC-MS analysis of the residual *n*-alkane. The mean values and standard deviations are shown. *, *p* < 0.05 compared with pCom8 control (*n* = 3/group).

To further elucidate the functions of the two *alkB* genes in strain CH91, several efforts were made to complement the individual *alkB1* and *alkB2* CH91 mutants, however, we could not find a suitable expression vector to develop the complementing assays. Therefore, *alkB1* and *alkB2* genes were cloned into the vector pCom8 and expressed in *P. fluorescens* KOB2Δ1. The recombinants KOB2Δ1 (pCom8/CH91*alkB1*) and KOB2Δ1 (pCom8/CH91*alkB2*) showed much better growth than the control strain KOB2Δ1 (pCom8) when grown on long-chain *n*-alkane mixture (Figure 4). Alkane degradation analysis revealed that the recombinant KOB2Δ1 (pCom8/CH91*alkB1*) degraded more C16–C26 *n*-alkanes and the recombinants KOB2Δ1 (pCom8/CH91*alkB2*) showed an increased degradation on C16–C30 *n*-alkanes when compared to those control strains harboring vector only (Figure 4). AlkB1 and AlkB2 showed activity on the different chain length ranges of *n*-alkane in KOBΔ1 from that in CH91. Possible reasons may be the presence of (i) a different regulatory mechanism responsible for longer-chain alkanes between KOB2Δ1 and CH91; (ii) an unknown factor(s) in the alkane hydroxylase system of CH91; (iii) a limitation in the uptake/catabolism of C32–C36 alkanes in strain KOB2Δ1. Smits et al. reported that *P. fluorescens* KOB2Δ1 is no longer able to grow on C12 to C16 alkanes but could grow on C18–C28 *n*-alkanes [15]. However, these results could indicate that CH91AlkB1 and CH91AlkB2 can degrade long-chain *n*-alkanes.

When the recombinants were grown on the *n*-alkane mixture, the degradation rate was reduced with the increasing chain length, which is in accordance with the results of two mutants (CH91Δ*alkB1* and CH91Δ*alkB2*) grown on the *n*-alkanes mixture. These data could indicate that the specific activity of CH91AlkB1 and CH91AlkB2 enzymes is reduced with increasing chain length, as it excludes the influence of different expression levels induced by different chain length *n*-alkane by using the *n*-alkanes mixture as the sole carbon source. Similar characteristics of alkane hydroxylase are also found in other studies [20,28,39]. In addition, KOB2Δ1 (pCom8/CH91*alkB2*) exhibited better growth and degradation ability than KOB2Δ1 (pCom8/CH91*alkB1*), which suggests the higher enzyme activity of CH91AlkB2 than CH91AlkB1.

The *alkB2* and *rubA1* genes and the *rubA1* and *rubA2* genes in the *alkB2* cluster of strain CH91 have overlapping stop and start codons, which indicates the translational coupling of *rubA1* and *rubA2* to *alkB2* for the production of stoichiometric amounts of the involved proteins and the important role of RubA1 and RubA2 for the function of AlkB [14]. We also tested the function of the two *rubA* genes by heterologous expression in KOB2Δ1. The *alkB2-rubA1-2* gene coupling fragment was directly amplified by PCR and cloned into the plasmid pCom8. The *alkB1-rubA1-2* gene coupling fragment was constructed by replacing *alkB2* in the *alkB2-rubA1-2* gene coupling fragment with the *alkB1* gene using the Gibson assembly method [29]. The recombinants KOB2Δ1 (pCom8/CH91*alkB1-rubA1-2*) and KOB2Δ1 (pCom8/CH91*alkB2-rubA1-2*) showed similar growth but higher *n*-alkane degradability in comparison with the recombinants KOB2Δ1 (pCom8/CH91*alkB1*) and KOB2Δ1 (pCom8/CH91*alkB2*), respectively (Figure 4). The growth and *n*-alkane degradation was not restored by the pCom8-*rubA1-2* plasmid carrying just only the two *rubA* genes. The results indicate that the co-expression of the coupling rubredoxins can enhance the alkane hydroxylation activities of CH91AlkB1 and CH91AlkB2 in *P. fluorescens* KOB2Δ1 but cannot broaden the chain length range of *n*-alkane degradation. This is in accordance with the previous result of Nie et al. as determined in *Dietzia* sp. DQ12-45-1b. They identified the rubredoxin domain as being necessary for the hydroxylation of long-chain *n*-alkanes with chain lengths ranging from C18 to C32 [39].

## 4. Conclusions

This study presented experimental evidence for elucidating the functional role of AlkB-type hydroxylase in the degradation of a broad range of *n*-alkanes. Combining the results of the transcriptional expression, knockout mutation, and heterologous expression of *n*-alkane hydroxylase genes (*alkB1* and *alkB2*) from strain CH91, it can be concluded that both novel AlkB1 and AlkB2 hydroxylases are responsible for C16–C36 *n*-alkanes’ degradation in *Rhodococcus* sp. CH91 and AlkB2 appeared to play a more important role than AlkB1. The results of the recombinants made in *Pseudomonas* and the mutants in *Rhodococcus* grown on the *n*-alkane mixture indicate that the enzyme activities of CH91AlkB1 and CH91AlkB2 are reduced with increasing chain length. The different profiles of the transcriptional expression as well as the *n*-alkane degradation of *alkB1* and *alkB2* in response to different *n*-alkane substrates suggest the expression of *alkB1* and *alkB2* follows different regulatory mechanisms. The experimental results substantially contribute to understanding the metabolic pathway of the long-chain *n*-alkane biodegradation in *Rhododcoccus* sp. CH91, and also provide potential gene candidates for engineering the bacteria used for bioremediation of petroleum hydrocarbon polluted sites.

## Data Availability

The GenBank/EMBL/DDBJ accession numbers for the sequences reported in this paper are OM831274 for the *Rhodococcus* sp. strain CH91 *alkB1* gene, OM831275 for the *Rhodococcus* sp. strain CH91 *alkB2* region.

## Supplementary Materials

The following supporting information can be downloaded at: https://www.mdpi.com/article/10.3390/microorganisms11061537/s1, Table S1: Bacterial strains and plasmids used in this study; Table S2: Primers used in this study.
