# Supplementary material for: Functional Analysis of Novel alkB Genes Encoding Long-Chain n-Alkane Hydroxylases in Rhodococcus sp. Strain CH91
Source: Microorganisms. 2023 Jun 9;11(6):1537. doi: 10.3390/microorganisms11061537 (PMC10304049; doi:10.3390/microorganisms11061537)
Supplement: Supplementary file 1 [file microorganisms-11-01537-s001.zip › microorganisms-2417009-supplementary.pdf]

Functional Analysis of Novel *alkB* Genes Encoding Long-Chain *n*-Alkane Hydroxylases in *Rhodococcus* sp. Strain CH91

Wei Xiang, Shan Hong, Yanfen Xue * and Yanhe Ma

State Key Laboratory of Microbial Resources, Institute of Microbiology, Chinese Academy of Sciences, Beijing 100101, China

* Correspondence: xueyf@im.ac.cn; Tel.: +86-10-64807618

Table S1 Bacterial strains and plasmids used in this study

Strain or plasmid	Relevant characteristics (genotype)	Reference or source
Strains		
P. fluorescens KOB2Δ1	*alkB1* knockout; C12-C16 Alk^{-}; C18-C28 Alk^{+}	[15]
KOB2Δ1(pCom8)	KOB2Δ1 harboring plasmid pCom8	[27]
KOB2Δ1(pCom8-CH91*alkB1*)	KOB2Δ1 harboring plasmid pCom8-CH91*alkB1*	This study
KOB2Δ1(pCom8-CH91*alkB2*)	KOB2Δ1 harboring plasmid pCom8-CH91*alkB2*	This study
KOB2Δ1(pCom8-CH91*alkB1-rub*)	KOB2Δ1 harboring plasmid pCom8-CH91*alkB1-rub*	This study
KOB2Δ1(pCom8-CH91*alkB2-rub*)	KOB2Δ1 harboring plasmid pCom8-CH91*alkB2-rub*	This study
Rhodococcus sp. CH91	wild type; grow on C16-C36	[22]
CH91Δ*alkB1*	CH91 with *alkB1* gene knockout; *alkB1*$^{-}$	This study
CH91Δ*alkB2*	CH91 with *alkB2* gene knockout; *alkB2*$^{-}$	This study
E. coli DH5α	strain used for general cloning	TransGen, China
Plasmid (cloning and expression vectors)		
pCom8	Broad host range expression vector; P*alkB*, Gm^{r}, *oriT*, and *alkS*	[27]
pCom8-CH91*alkB1*	pCom8 with *alkB1* gene from *Rhodococcus* sp. CH91	This study
pCom8-CH91*alkB2*	pCom8 with *alkB2* gene from *Rhodococcus* sp. CH91	This study
pCom8-CH91*alkB1-rub*	pCom8 with *alkB1-rub* genes from *Rhodococcus* sp. CH91	This study
pCom8-CH91*alkB2-rub*	pCom8 with *alkB2-rub* genes from *Rhodococcus* sp. CH91	This study
Plasmid (knockout vectors)		
pNV-Pa2-Cas9	pNV18.1 derivate, codon-optimized cas9 under promoter Pa2, KmR	[26]
pRCTc-Pa2-Che9c60&61	pRCTc derivate, *che9c60&61* under promoter Pa2, Tc^{R}	[26]
pBNVCm-BbsI-sgRNA	pBNVCm derivate, empty sgRNA cassette under promoter PamiC	[26]
pBNVCm-alkB1-sgRNA	pBNVCm derivate, sgRNA targeting *alkB1*	This study
pBNVCm-alkB2-sgRNA	pBNVCm derivate, sgRNA targeting *alkB2*	This study

Table S2 Primers used in this study

Gene	Primer	Sequence(5'-3')
Primers for RT-qPCR*		
16S rRNA	16S-up	TGGAATGCTGGGCGATGC
	16S-down	TGCGGGAAAGGTTGATGC
alkB1	alkB1-up	TGTTCCAACCTGTTCCTCT
	alkB1-down	GATCAGTACCGCATATCCG
alkB2	alkB2-up	CCTGGTGACGAACATCTTC
	alkB2-down	AATGTAGGCGAGGACGAT
Primers for gene knockout in strain CH91		
alkB1	*alkB1*-up-F	GAGTAACCCTGCGCATTGGGCA
	alkB1-up-R	GGACCGGACCAACACCCATCCCCCATA
	alkB1-down-F	TGGGTGTTGGTCCGGTCCACAGGCA
	alkB1-down-R	TCCGCCCGTCGAATTCGC
alkB1-sgRNA	*alkB1*-sgRNA-F	ATGCGAAGACATAACGTTCCTGCCACGCACCGTCTTGTTTCGGTCTTCATGC
	alkB1-sgRNA-R	GCATGAAGACCGAAACAAGACGGTGCGTGGCAGGAACGTTATGTCTTCGCAT
alkB2	*alkB2*-up-F	AACGAACGTGACTTCGTTCCG
	alkB2-up-R	AGCCGCACCACCGTCGGAGGTGCCGC
	alkB2-down-F	CGACGGTGGTGCGGCTTTGACGAGCAC
	alkB2-down-R	TTGAAATCGAGACCGTTGATCCG
alkB2-sgRNA	*alkB2*-sgRNA-F	ATGCGAAGACATAACGGTGAACTATCTCGAGCACTAGTTTCGGTCTTCATGC
	alkB2-sgRNA-R	GCATGAAGACCGAAACTAGTGCTCGAGATAGTTCACCGTTATGTCTTCGCAT
Primers for heterologous expression in *P. fluorescens* KOB2Δ1		
alkB1	*alkB1*-F	TGGAGAATTCCATATGGTGGATTCGGCGACTCCTCG
	alkB1-R	GCCAAAACAGAAGCTTCTACGCTGCCGACGTGTAG
alkB2	*alkB2*-F	ATTTAATAAAAATTGGAGAATTCCATATGATGAGGAGACCCGACATGAG
	alkB2-R	CCCAAGCTTTCACCGACCTTCCGCAGCAG
rub	*rub*-F	ATTTAATAAAAATTGGAGAATTCCATATGGTGACCGCGATGTCCGC
	rub-R	CATCCGCCAAAACAGAAGCTTTCACCGGCGGACGATTTCC
alkB1-rub	*alkB1-rub*-F	TCCATATGGATTCGGCGACTCCTCGCT
	alkB1-rub-R	GACGACCCGTAGCGGGCGAGCAGGG
alkB2-rub	*alkB2-rub*-F	ATTTAATAAAAATTGGAGAATTCCATATGATGAGGAGACCCGACATGAG
	alkB2-rub-R	CCCAAGCTTTCACCGGCGGACGATTTCCA

* The amplification efficiencies (E) of the primers used in the RT-qPCR were 2.065, 1.833 and 1.956, respectively, for 16S rRNA, *alkB1* and *alkB2* genes.
